# Importance of physicians’ attire: factors influencing the impression it makes on patients, a cross-sectional study

**DOI:** 10.1186/1447-056X-13-2

**Published:** 2014-01-08

**Authors:** Hiroshi Kurihara, Takami Maeno, Tetsuhiro Maeno

**Affiliations:** 1 Department of General Medicine and Primary Care, University of Tsukuba Hospital, Amakubo 2-1-1, Tsukuba City, Ibaraki Prefecture 305-0005, Japan

**Keywords:** Doctor-patient relationship, Confidence in doctors, Patients’ preferences, White coat, Scrubs, Doctor’s attire, Communication

## Abstract

**Objective:**

The aim of the present study was to determine the importance of physician attire in inspiring confidence in patients, patient preferences and factors influencing the impression made by the clothing worn by doctors.

**Methods:**

Self-administered questionnaires were distributed and completed in five pharmacies across Japan (April–October 2012) to patients or their carers (aged ≥20 years). The survey was performed over 2 consecutive days in each pharmacy. To estimate patient confidence in doctors, questions were asked addressing six items, namely doctors’ attire, speech (way of speaking, volume, tone etc.), age, gender, title (professor, PhD etc.) and reputation. Participants were shown photographs of five different types of attire for male and female doctors (i.e. white coats, scrubs, semiformal, smart casual and casual wear) and asked to rate the appropriateness of each clothing style using a five-point Likert scale.

**Results:**

Of the 1411 patients or carers who attended the pharmacies, 530 responded to the questionnaire, with 491 complete responses used in subsequent analyses. The mean age of respondents was 51.9 years and 40.3% were male. Speech was the most important factor (mean score 4.60) in determining confidence in doctors, followed by reputation (4.06) and attire (4.00). With regard to attire, regardless of a doctor’s gender, the white coat was judged to be the most appropriate style of dress, followed by surgical scrubs. Only the preference for scrubs was significantly affected by age, gender and region (*P* < 0.05). Using binomial logistic regression analysis, we evaluated the effects of age on the appropriateness (Likert score 3–5) versus inappropriateness (score 1–2) of scrubs. There was a significant increase in the number of subjects aged 50–64 and >65 years of age who thought scrubs were inappropriate compared with those aged 20–34 years (adjusted odds ratios of 4.30 and 12.7 for male doctors, and 3.66 and 6.91 for female doctors).

**Conclusions:**

Attire is one of the important factor that inspires patient confidence in physicians. White coats were deemed the most appropriate clothing style for doctors, followed by scrubs. However, older participants perceived scrubs to be less appropriate attire than younger subjects.

## Background

The patient–doctor relationship is considered the cornerstone of medical care. Physician attire is not only a way of protecting oneself from microscopic organisms, but is also a symbol of competence and status. Previous studies have shown that patients regard the way in which physicians dress as important. Yamada et al. reported that approximately 70% of their study participants considered that physicians’ attire influenced their confidence in the physicians [1]. However, others have reported that patient satisfaction is unaffected by the way in which doctors dress [2,3]. Other factors, such as age, gender and the way in which physicians speak, have been reported as influencing patient trust [4,5]. However, few studies have investigated the importance of doctors’ attire relative to other factors inspiring confidence.

Physicians once only wore white coats, but recently their dress has become more varied. Along with traditional long-sleeved white coats, doctors now appear more casually dressed. Some studies report that patients tend to prefer that doctors wear white coats [1,6-8], whereas others have reported that patients do not mind what doctors wear [9,10] or prefer that they wear semiformal or formal clothing rather than white coats [11,12]. Because most previous studies have been conducted in a single hospital or clinic [1,6-9,12], the results may reflect the culture of a specific setting and its influence on patients. The contribution of other factors (patients’ age, gender, area) on the impression created by what doctors wear is not evident.

Demonstrating the importance of physician attire and the factors that may influence patient responses will allow doctors to pay more attention to their clothing to foster good patient–doctor relationships. Preferences regarding physician attire will change with time, and so newer information is currently needed. The aim of the present study was to address the following three questions:

1. Compared with other factors, how important is physician attire in contributing to patient confidence?

2. What types of physician attire do patients prefer?

3. What factors influence patients’ impressions of physicians’ attire?

## Methods

### Study design

The present cross-sectional study used self-administered questionnaires. The study was approved by the Ethics Committee of the Faculty of Medicine of the University of Tsukuba.

### Study setting

The survey was conducted at five pharmacies in three regions in Japan, namely Ibaraki Prefecture (Region 1; two pharmacies), Niigata Prefecture (Region 2; two pharmacies) and Tokyo (Region 3; one pharmacy). There were two reasons why we chose pharmacies. First, they allowed for a wider sample, because patients could be recruited in the one pharmacy who consulted more than one hospital or clinic. Second, by recruiting patients in pharmacies, we thought they would be able to answer the questionnaire without worrying about surveillance from medical personnel.

#### Participants

Patients or their carers (≥20 years of age at the time of the study) attending the pharmacies were recruited as study participants. Written informed consent was obtained from the participants prior to taking the questionnaire. Patients or their carers who declined complete the questionnaire or were too ill to answer the questions were excluded from the study.

#### Survey period

The survey was performed over 2 consecutive days in each pharmacy in the period April–October 2012.

#### Questionnaire

To develop the questionnaire, we reviewed previous studies, discussed them among the researchers, and then decided on the questionnaire items. For factors that might influence the doctor’s confidence, we chose items reported in previous studies as important [1,4,5,13]. Finally we investigated six items, namely attire, speech (way of speaking, volume, pitch etc.), age, gender, title (professor, PhD etc.) and the doctor’s reputation. Regarding to reputation, we would like to explore the impact of doctor’s impression from people around such as doctor’s character, communication style and skills. Participants were asked to rank the importance of each factor on a five-point Likert scale, from 1 (not important at all) to 5 (very important).

**Figure 1:**
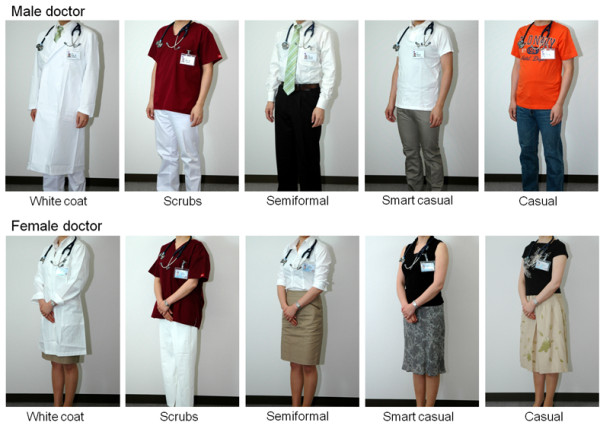
**Photographs shown to study participants of the different clothing styles for male and female doctors.** The “white coat” style consisted of a shirt, necktie and white coat for men, and a skirt and white coat for women; the scrubs consisted of dark red scrubs for both men and women; semiformal clothing consisted of a shirt and necktie for men, and a blouse and skirt for women; smart casual clothing was a white polo shirt for men, and a sleeveless blouse and skirt for women; casual clothing consisted of jeans and a T-shirt for men, and a T-shirt with skirt for women. In all photographs, the background, camera angle, posture, position of the stethoscope and name tag were kept constant, and, to avoid any effects of expression and countenance, no faces appeared in any of the photographs.

For the doctor’s attire, according to previous studies, we chose five clothing styles (white coat, scrubs, semiformal, smart casual and casual dress) and showed them to the participants by photographs [1,9,12]. Participants were shown two sets of five photographs (Figure 1), one set showing clothing styles for male doctors and the other showing styles for female doctors. Briefly, the “white coat” style consisted of a shirt, necktie and white coat for men, and a skirt and white coat for women; the scrubs consisted of dark red scrubs for both men and women; semiformal clothing consisted of a shirt and necktie for men, and a blouse and skirt for women; smart casual clothing was a white polo shirt for men, and sleeveless blouse and skirt for women; and casual clothing consisted of jeans and a T-shirt for men, and a T-shirt with skirt for women.In all photographs, the background, camera angle, posture, position of the stethoscope and name tag were kept constant. To avoid potential effects of expression and countenance, no faces appeared in any of the photographs. These photographs were presented to participants on a tablet. Participants were asked to indicate the appropriateness of the attire using a five-point Likert scale, from 1 (absolutely disagree) to 5 (absolutely agree).

Finally, background information regarding the age and gender of the respondents was collected.

#### Statistical analysis

Each of the items evaluated using the five-point Likert scale was given an overall score from 1 (“not important” at all or “absolutely disagree”) to 5 (“very important” or “absolutely agree”). As in a previous study [14], we divided the participants into four, 15-year age-range groups: (i) <35 years of age (younger group); (ii) from 35 to 49 years of age (lower-middle group); (iii) from 50 to 64 years of age (higher-middle group); and (iv) >65 years of age (elderly group).

Data were analysed as follows. First, to analyse the factors influencing confidence in doctors, the mean score was calculated for each item. Then, univariate analysis was used to analyse the factors associated with preferences for each style. A *t*-test was used to determine the effects of gender on factors affecting preferences, whereas analysis of variance (ANOVA) was used to investigate the effects of age and region in Japan.

A multivariate model was used to determine what was considered inappropriate physician attire. In this analysis, clothing styles were divided into an “inappropriate” group (= 1) based on Likert scores of 1–2 and into an “appropriate” group (= 0) based on scores of 3–5, with these groups being defined as the dependent variables in binomial logistic regression analysis. The independent variables were gender, age group and region. The reference groups for the individual factors were male gender, age <35 years (younger group) and Region 1. Odds ratios (OR) and 95% confidence intervals (CI) were calculated to estimate the association between inappropriateness and the different factors.

Where appropriate, Likert scores for the different items are given as the mean ± SD. All analyses were conducted using SPSS, version 21.0 J. In all analyses, *P* < 0.05 was considered significant.

## Results

In all, 1411 patients or their carers attended one of the pharmacies, with 530 patients or their carers agreeing to participate in the study (37.6% response rate). Of the 530 people who agreed to participate in the study, complete survey responses were available for 491 and these were subsequently used in the analyses. The characteristics of the study participants are listed in Table 1.

**Table 1:** Participant characteristics (n = 491)

	**No. participants**	**% men**	**Mean (±SD) age (years)**	**No. participants in different age groups**			
				**20–34 years**	**35–49 years**	**50–64 years**	**65+ years**
Region 1	192	43.2	51.7 ± 15.9	35	49	63	45
Region 2	189	40.7	56.6 ± 14.4	16	32	86	55
Region 3	110	34.5	44.3 ± 13.3	31	41	31	7
Total	491	40.3	51.9 ± 15.6	82	122	180	107

The survey was conducted at five pharmacies in three regions in Japan, namely Ibaraki Prefecture (Region 1; two pharmacies), Niigata Prefecture (Region 2; two pharmacies) and Tokyo (Region 3; one pharmacy).

**Figure 2:**
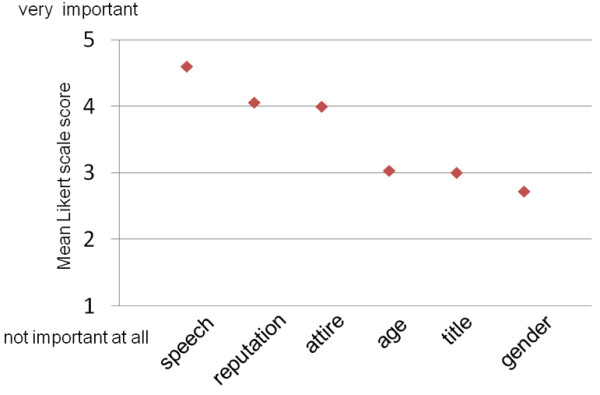
**Effects of six items on patient confidence in doctors.** Results show the mean scores on a five-point Likert scale for the importance of speech, reputation, attire, age, title and gender on patient confidence in doctors.

Figure 2 shows the influence of the six items evaluated in the present study on patient confidence in doctors. Speech (mean [±SD] score 4.60 ± 0.68), reputation (4.06 ± 0.93) and attire (4.00 ± 0.94) were considered important by patients. Less crucial factors were doctors’ titles (3.04 ± 1.01), age (3.01 ± 1.04) and gender (2.73 ± 1.01).

White coats were considered to be the most appropriate style of clothing, following by scrubs, for both male and emale physicians. The other clothing styles (i.e. semiformal, smart casual and causal) were given lower scores. Only 5.3% and 1.0% of participants thought that white coats were inappropriate (score of 1 or 2) for male and female doctors, respectively. A similar proportion of participants thought scrubs were inappropriate for male and female doctors (15.5% *vs* 15.1%, respectively). However, a higher proportion of participants thought that semiformal, smart casual and casual dress was inappropriate for both male (48.5%, 23.8% and 73.3%, respectively) and female (37.1%, 73.1% and 79.8%, respectively) physicians.

There were significant differences only in mean scores for scrubs as a clothing style among the three regions (Table 2). In each of the three regions, white coats were considered to be the most appropriate clothing style for both male and female doctors, followed by scrubs and smart casual clothing for men and scrubs alone for women.

**Table 2:** Evaluation of the appropriateness of different types of attire according to region in Japan and age group

	**Region in Japan**				**Age group (years)**				
	**Region 1**	**Region 2**	**Region 3**	***P*-value**	**20–34**	**35–49**	**50–64**	**65+**	***P*-value**
**Male physicians**									
White coat	4.17 ± 0.94	4.10 ± 0.93	4.25 ± 0.89	0.38	4.32 ± 0.77	4.18 ± 0.86	4.04 ± 0.94	4.22 ± 1.06	0.12
Scrubs	3.66 ± 1.04	3.35 ± 1.10	3.58 ± 0.96	0.01	3.90 ± 0.86	3.75 ± 0.89	3.46 ± 1.04	3.08 ± 1.21	<0.01
Semiformal	2.58 ± 0.96	2.56 ± 1.05	2.66 ± 0.94	0.68	2.67 ± 0.90	2.57 ± 0.94	2.48 ± 0.96	2.74 ± 1.14	0.17
Smart casual	3.24 ± 1.12	3.22 ± 1.13	3.51 ± 1.01	0.07	3.35 ± 1.16	3.22 ± 1.01	3.18 ± 1.08	3.53 ± 1.18	0.50
Casual	1.96 ± 0.84	2.08 ± 1.00	1.89 ± 0.87	0.17	1.85 ± 0.79	1.87 ± 0.79	1.97 ± 0.92	2.28 ± 1.06	0.02
**Female physicians**									
White coat	4.38 ± 0.81	4.28 ± 0.78	4.32 ± 0.81	0.51	4.51 ± 0.65	4.30 ± 0.77	4.25 ± 0.80	4.35 ± 0.90	0.10
Scrubs	3.79 ± 1.04	3.35 ± 1.06	3.69 ± 0.99	<0.01	3.99 ± 0.84	3.87 ± 0.94	3.49 ± 1.05	3.16 ± 1.14	<0.01
Semiformal	2.89 ± 1.01	2.76 ± 1.01	2.94 ± 1.05	0.29	3.01 ± 0.95	2.80 ± 0.90	2.73 ± 1.01	2.98 ± 1.13	0.08
Smart casual	2.09 ± 0.92	2.02 ± 0.96	2.11 ± 0.92	0.68	2.15 ± 0.86	2.02 ± 0.87	1.98 ± 0.88	2.21 ± 1.12	0.19
Casual	1.93 ± 0.87	1.90 ± 0.93	1.82 ± 0.87	0.55	1.80 ± 0.79	1.86 ± 0.81	1.86 ± 0.84	2.06 ± 1.04	0.17

Data show the mean ± SD scores after participants had been asked to indicate the appropriateness of the attire shown in Figure 1 using a five-point Likert scale, ranging from 1 (absolutely disagree) to 5 (absolutely agree).

The survey was conducted at five pharmacies in three regions in Japan, namely Ibaraki Prefecture (Region 1; two pharmacies), Niigata Prefecture (Region 2; two pharmacies) and Tokyo (Region 3; one pharmacy).

Analysis of variance (ANOVA) was used for univariate analysis.

Table 2 also lists mean scores for the each of the five clothing styles according to age group. Participants ranked the white coat style as the best for doctors of both genders. For some attire, the mean scores for the different clothing styles varied among the different age groups. Significant differences were found for the ranking of scrubs (*P* < 0.01) and casual attire (*P* < 0.01) for male doctors, and for scrubs for female doctors (*P* < 0.01). Elderly people considered scrubs less appropriate for both male and female doctors. Regardless of physician gender, scrubs were rated significantly more highly by female participants, whereas male participants ranked the smart casual style more highly for female doctors.

Significant differences according to age group, region and gender were evident only for scrubs. A multivariate model was fitted to the factors, with Table 3 listing the OR for the inappropriateness of scrubs according to each factor after adjustment for all others. The main finding is a significant increase in the inappropriateness of scrubs according to participants aged 50–64 and >65 years compared with the younger group, with an adjusted OR of 4.30 (95% CI 1.24–14.90) and 12.7 (95% CI 3.64–44.8) for male doctors, respectively, and 3.66 (95% CI 1.22–11.0) and 6.91 (95% CI 2.24–21.33) for female doctors, respectively.

**Table 3:** Association between subject characteristics and the perceived “inappropriateness” of scrubs as clothing for male and female doctors

**Factor**	**Male doctors**		**Female doctors**	
	**OR (95% CI)**	***P*-value**	**OR (95% CI)**	***P*-value**
Gender				
Male	1.00		1.00	
Female	0.77 (0.45–1.30)	0.33	0.67 (0.40–1.14)	0.14
Area in Japan				
Region 1	1.00		1.00	
Region 2	1.78 (0.99–3.21)	0.05	1.98 (1.10–3.58)	0.02
Region 3	1.54 (0.69–3.41)	0.29	1.56 (0.70–3.47)	0.27
Age group (years)				
20–34	1.00		1.00	
35–49	2.11 (0.55–8.07)	0.28	1.38 (0.40–4.77)	0.61
50–64	4.30 (1.24–14.90)	0.02	3.66 (1.22–10.98)	0.02
65+	12.77 (3.64–44.76)	<0.01	6.91 (2.24–21.33)	<0.01

The survey was conducted at five pharmacies in three regions in Japan, namely Ibaraki Prefecture (Region 1; two pharmacies), Niigata Prefecture (Region 2; two pharmacies) and Tokyo (Region 3; one pharmacy).

In this analysis, scrub styles were divided into an “inappropriate” group (= 1) based on Likert scores of 1–2 and into an “appropriate” group (= 0) based on scores of 3–5, with these groups being defined as the dependent variables in binomial logistic regression analysis. The independent variables were gender, age group and region. The reference groups for the individual factors were male gender, age <35 years (younger group) and Region 1.

*OR* odds ratio, *CI* confidence interval.

There was also a significant difference among regions in the appropriateness of scrubs, with a significant increase in appropriateness reported for female doctors in Region 2 compared with Region 1 (adjusted OR of 1.98; 95% CI 1.10–3.58).

## Discussion

In terms of inspiring confidence and trust, participants rated a doctor’s speech, reputation and attire as quite important, but age, title and gender less so. However, each factor may make an important contribution to a patient’s judgment of his/her doctor. Shah and Ogden reported that patients consider age and gender to be important because these factors relate to personal manners, as well as technical and explanatory skills [4]. In another study, the tone of voice was significantly associated with malpractice claim history [5], whereas Torres et al. found that a physician’s reputation is positively associated with patient trust and satisfaction [13]. Many studies have investigated factors affecting patient faith in doctors. However, few studies have been comparative. In the present study, we show the importance of attire in relation to other factors. The importance of attire is not inferior, but actually rather high.

Our finding that patients tend to prefer that doctors wear white coats is similar to that reported by many other studies worldwide [1,6,7,15-19]. Despite differences between countries, the white coat is a symbol of doctors, and it may create a sense of ease or confidence in patients. In the present study, only 5% of participants regarded the white coat as inappropriate. Considering that physician attire is important to patients, white coats are an appropriate clothing style for doctors.

In the present study, almost 40% of participants considered a semiformal style, which is often seen in Japanese hospitals, as less appropriate. The preference for this clothing style has varied among studies [1,6,14]. For example, Lill et al. reported that the semiformal was the favourite style in New Zealand [12], yet Yamada et al. found that patients do not like this style of clothing in Japan [1]. The findings of Yamada et al. were echoed in the present study in other regions in Japan.

Patients tended to think that scrubs were almost as appropriate as white coats, but the inappropriateness of scrubs differed according to age and regions. Elderly people regarded scrubs as less appropriate than did younger people. The same finding has been reported in the UK [6]. Doctors could consider this when attending elderly patients. With regard to the difference between regions, this could reflect different levels of recognition of scrubs, which are relatively new attire for doctors, although identifying such reasons was beyond the scope of the study design.

In 2007, the British Department of Health published guidelines for good practice regarding uniforms and work wear [20]. Because cuffs become heavily contaminated and are more likely to come into contact with patients, the guidelines recommend wearing short-sleeved shirts or blouses and to avoid wearing white coats when providing patient care [20]. Surgical scrubs are more hygienic and comfortable for doctors; thus, they may become more common attire in the future. In this case, it may be necessary to inform elderly patients of the medical virtues of this type of clothing.

### Limitations

There are several limitations to the present study. First, the response rate was only 35% and so there may be a problem with internal validity. However, the results (i.e. white coats and scrubs are the preferred clothing style) are similar to those reported in a previous study conducted in Japan [1]. Thus, we think the reliability of the study is acceptable.

Second, we are unsure whether the pharmacies chosen for subject recruitment accurately represent the regions in which they are located or even all areas of Japan. Settings (inpatient vs outpatient), cultural differences and physician specialties may limit the applicability of our results regarding the preferences for doctors’ attire. However, the survey was conducted in three, not just one, regions of Japan to collect a wider sample.

Third, the colour of the scrubs (dark red) in the present study may have contributed to the generational reaction to them, with different colours perhaps eliciting different reactions. So, using only one colour for the scrubs is also a limitation of the study. Patients, especially elderly patients, may have found it odd that doctors were not wearing white clothing, although scrub colours vary. Because younger people have seen colourful scrubs in TV dramas, such as “ER”, they may not find the red scrubs incongruous. Thus, it may be necessary to clarify the relationship between patient age and their preference for the colour of physicians’ attire.

The present study has demonstrated the importance of physician attire in patient perceptions. However, the influence of a particular style of attire may last only a short time. Thus, it is necessary to clarify whether clothes can influence long-term patient–doctor relationships.

## Conclusions

We have shown physicians attire after the speech and reputation is one of the most important factor inspiring physician confidence in their physicians. We have shown the importance of physicians’ attire in patient perceptions. Specifically, it is an important factor inspiring physician confidence. White coats were seen as the most appropriate style for doctors, regardless of gender, followed by scrubs. However, older study participants perceived scrubs as less appropriate attire than younger participants.

## Competing interests

The authors declare that they have no competing interests.

## Authors’ contributions

HK participated in the proposal’s design, coordination, and data collection, carried out the study and drafted and completed the manuscript. Takami M participated in the proposal’s design, and coordination, and in drafting the manuscript. Tetsuhiro M participated in the proposal’s design, and coordination, and in drafting the manuscript. All authors read and approved the final manuscript.

## References

[B1] Yamada Y Takahashi O Ohde S Deshpande GA Fukui T Patients’ preferences for doctors’ attire in Japan Intern Med 2010 13 1521 1526 10.2169/internalmedicine.49.3572 20686283

[B2] Druss RG The magic white coat Ann Intern Med 1998 13 743 10.7326/0003-4819-129-9-199811010-00014 9841610

[B3] Hennessy N Harrison DA Aitkenhead AR The effect of the anaesthetist’s attire on patient attitudes. The influence of dress on patient perception of the anaesthetist’s prestige Anaesthesia 1993 13 219 222 10.1111/j.1365-2044.1993.tb06905.x 8460799

[B4] Shah R Ogden J ‘What’s in a face?’ The role of doctor ethnicity, age and gender in the formation of patients’ judgements: an experimental study Patient Educ Couns 2006 13 136 141 10.1016/j.pec.2004.12.005 16442455

[B5] Ambady N Laplante D Nguyen T Rosenthal R Chaumeton N Levinson W Surgeons’ tone of voice: a clue to malpractice history Surgery 2002 13 5 9 10.1067/msy.2002.124733 12110787

[B6] Gherardi G Cameron J West A Crossley M Are we dressed to impress? A descriptive survey assessing patients’ preference of doctors’ attire in the hospital setting Clin Med 2009 13 519 524 10.7861/clinmedicine.9-6-519 20095290 PMC4952286

[B7] Rehman SU Nietert PJ Cope DW Kilpatrick AO What to wear today? Effect of doctor’s attire on the trust and confidence of patients Am J Med 2005 13 1279 1286 10.1016/j.amjmed.2005.04.026 16271913

[B8] Cha A Hecht BR Nelson K Hopkins MP Resident physician attire: does it make a difference to our patients? Am J Obstet Gynecol 2004 13 1484 1488 10.1016/j.ajog.2004.02.022 15167876

[B9] Shelton CL Raistrick C Warburton K Siddiqui KH Can changes in clinical attire reduce likelihood of cross-infection without jeopardising the doctor-patient relationship? J Hosp Infect 2010 13 22 29 10.1016/j.jhin.2009.07.031 19914736

[B10] Boon D Wardrope J What should doctors wear in the accident and emergency department? Patients’ perception J Accid Emerg Med 1994 13 175 177 10.1136/emj.11.3.175 7804584 PMC1342426

[B11] McKinstry B Wang JX Putting on the style: what patients think of the way their doctor dresses Br J Gen Pract 1991 13 270 275–278 1747264 PMC1371685

[B12] Lill MM Wilkinson TJ Judging a book by its cover: descriptive survey of patients’ preferences for doctors’ appearance and mode of address BMJ 2005 13 1524 1527 10.1136/bmj.331.7531.1524 16373739 PMC1322253

[B13] Torres E Vasquez-Parraga AZ Barra C The path of patient loyalty and the role of doctor reputation Health Market Q 2009 13 183 197 10.1080/07359680903263565 19813122

[B14] Hueston WJ Carek SM Patients’ preference for physician attire: a survey of patients in family medicine training practices Fam Med 2011 13 643 647 22002776

[B15] Gooden BR Smith MJ Tattersall SJ Stockler MR Hospitalised patients’ views on doctors and white coats Med J Aust 2001 13 219 222 11587285 10.5694/j.1326-5377.2001.tb143103.x

[B16] Turner RN Leach J Robinson D First impressions in complementary practice: the importance of environment, dress and address to the therapeutic relationship Complement Ther Clin Pract 2007 13 102 109 10.1016/j.ctcp.2006.10.001 17400145

[B17] Rowland PA Coe NP Burchard KW Pricolo VE Factors affecting the professional image of physicians Curr Surg 2005 13 214 219 10.1016/j.cursur.2004.08.008 15796943

[B18] Douse J Derrett-Smith E Dheda K Dilworth JP Should doctors wear white coats? Postgrad Med J 2004 13 284 286 10.1136/pgmj.2003.017483 15138319 PMC1743003

[B19] Keenum AJ Wallace LS Stevens AR Patients’ attitudes regarding physical characteristics of family practice physicians South Med J 2003 13 1190 1194 10.1097/01.SMJ.0000077011.58103.C1 14696870

[B20] Uniforms and workwear:an evidence base for developing local policy http://www.dh.gov.uk/prod_consum_dh/groups/dh_digitalassets/documents/digitalasset/dh_078435.pdf

